# A systematic review of adherence in Indigenous Australians: an opportunity to improve chronic condition management

**DOI:** 10.1186/s12913-017-2794-y

**Published:** 2017-12-27

**Authors:** Jessica Langloh de Dassel, Anna P. Ralph, Alan Cass

**Affiliations:** 1 Charles Darwin University, Ellengowan Dr, Casuarina, Darwin, NT 0810 Australia; 2 Menzies School of Health Research, Bld 58, Royal Darwin Hospital Campus, Rocklands Drive, Tiwi, Darwin, NT 0811 Australia

**Keywords:** Indigenous health, Chronic disease, Adherence

## Abstract

**Background:**

Indigenous Australians experience high rates of chronic conditions. It is often asserted Indigenous Australians have low adherence to medication; however there has not been a comprehensive examination of the evidence. This systematic literature review presents data from studies of Indigenous Australians on adherence rates and identifies supporting factors and impediments from the perspective of health professionals and patients.

**Methods:**

Search strategies were used to identify literature in electronic databases and websites. The following databases were searched: Scopus, Medline, CINAHL Plus, PsycINFO, Academic Search Premier, Cochrane Library, Trove, Indigenous Health infonet and Grey Lit.org. Articles in English, reporting original data on adherence to long-term, self-administered medicines in Australia’s Indigenous populations were included.

Data were extracted into a standard template and a quality assessment was undertaken.

**Results:**

Forty-seven articles met inclusion criteria. Varied study methodologies prevented the use of meta-analysis. Key findings: health professionals believe adherence is a significant problem for Indigenous Australians; however, adherence rates are rarely measured. Health professionals and patients often reported the same barriers and facilitators, providing a framework for improvement.

**Conclusions:**

There is no evidence that medication adherence amongst Indigenous Australians is lower than for the general population. Nevertheless, the heavy burden of morbidity and mortality faced by Indigenous Australians with chronic conditions could be alleviated by enhancing medication adherence. Some evidence supports strategies to improve adherence, including the use of dose administration aids. This evidence should be used by clinicians when prescribing, and to implement and evaluate programs using standard measures to quantify adherence, to drive improvement in health outcomes.

**Electronic supplementary material:**

The online version of this article (10.1186/s12913-017-2794-y) contains supplementary material, which is available to authorized users.

## Background

Chronic conditions such as type 2 diabetes mellitus, hypertension and kidney disease impair the health of many Aboriginal Australian and/or Torres Strait Islander people (hereafter the term ‘Indigenous’ is used). In 2012–13, an estimated 20% of Indigenous Australian adults had high blood pressure, 25% had elevated cholesterol and 18% had indicators of chronic kidney disease [1]. Chronic conditions lead to considerable morbidity and premature mortality, contributing to 80% of the difference in life expectancy between Indigenous Australians and non-Indigenous Australians [2].

Management of chronic conditions usually requires a combination of behaviour modification and taking prescription medicines; accordingly effective management relies in part on medication adherence. The determinants of adherence to medicines are complex and incorporate human behaviour, health literacy and adequate access to resources to support adherence. Not surprisingly, numerous studies in the ‘general’ population indicate that when adherence is suboptimal health outcomes are poorer [3]. Indigenous Australians continue to die almost ten years earlier than non-Indigenous Australians [1]. The Pharmaceutical Society of Australia recently stated that inadequate medication adherence will impede improvements in the life expectancy for Indigenous Australians living with chronic conditions [4] and many health professionals and researchers assert that adherence in this population is especially challenging. Two thirds of Indigenous Australians have at least one chronic condition [1] but there has been limited objective examination of the contribution of adherence to medicines to chronic condition management in this population. Very few studies have quantified adherence for this population or examined the association between adherence and clinical outcomes for Indigenous Australians.

Adherence in the context of Indigenous health in Australia has been reviewed previously, though not systematically nor focusing on chronic conditions, and no attempts to assess the quality of the existing literature have been made. Davidson et al. identified cost of medications, patient mobility and ‘culturally alienating’ health services as barriers to adherence, and recommended adherence support strategies including regimen simplification, use of dose administration aids (DAAs) and building the cultural competence of health professionals to strengthen relationships with patients [5]. A review in the *Medical Student Journal of Australia* noted that inadequate family support and culturally inappropriate service delivery could all impair adherence [6]. Strategies suggested for supporting adherence included training health professionals in ‘cultural values and healthcare beliefs of Aboriginal and Torres Strait Islander communities’, increased use of interpreters and the subsidisation of medications for Indigenous Australians [6]. While many Indigenous Australians (35%) live in major city areas, the majority (44%) live in regional areas and 21% live in remote areas [7], so all health services in Australia need to provide culturally appropriate care.

In this era of escalating prevalence of chronic conditions, especially in populations of Indigenous Australians, clinicians and researchers require high-quality evidence to guide approaches to optimising adherence. The data on adherence rates for this population are often based on anecdote and information on appropriate strategies requires collation and analysis so that recommendations can be made.

Therefore we aimed to undertake a systematic literature review to provide a comprehensive compilation and examination of the literature on adherence to long-term medicines by Indigenous Australians living with chronic conditions. The primary objectives were: to synthesise data on the rates of adherence to medicines; explore health professionals’ attitudes towards adherence, and examine the impediments to and supporting factors of adherence as reported by health professionals and patients. The secondary objective was to identify and collate data on the health outcomes associated with adherence.

## Methods

The review was conducted and reported according to the PRISMA guidelines [8].

### Eligibility criteria

Studies and evaluations of any design reporting original data on adherence to self-administered medicines for chronic conditions were included. The authors were aware that very few randomised controlled trials had been conducted to examine this issue, so a deliberately broad inclusion criterion was applied regarding study methodology. The study population had to include Aboriginal and/or Torres Strait Islander Australians and results needed to be disaggregated by ethnicity. Eligible clinical trials and intervention studies were included in the facilitators and barriers section; only baseline adherence rates from these studies (where reported) were included in the compilation of adherence rate data since adherence during clinical trials is often higher than in real-world settings [9]. To maximise information available on health professionals’ perspectives commentary pieces by experts in the field were also included. Results were limited to articles published in English.

Most people with chronic conditions rely on self administered medications to manage their health, so studies reporting adherence to dialysis, chemotherapy and radiation therapy and directly administered medications (such as Benzathine Penicillin G injections) were excluded. Treatment and prophylaxis for tuberculosis were included as these treatment regimens are prolonged.

### Information sources and search strategy

Articles were identified through database searches, citation searching and review by experts. The electronic databases searched were: Scopus, Medline, CINAHL Plus, PsycINFO, Academic Search Premier, Cochrane Library, National Library of Australia (Trove) (limited to books and theses), Indigenous Health infonet, and Grey Lit.org, from inception until 23 February 2015. In addition the Charles Darwin University library catalogue was searched and a Google search was conducted (limited to sites ending in .gov.au or org.au, the first ten pages of results reviewed). A shortlist of relevant references was distributed to experts in the field who were asked to identify any missing publications. In addition the reference lists of included articles were searched.

The search strategy was kept intentionally broad to ensure all data relevant to chronic conditions were included. The search strategy for Medline was: (((*adheren* OR *complian* OR concord*) and (treatment* or medicine* OR medication* OR drug*)) or (MM “Patient Compliance+” OR MM “Medication Adherence”and (indigenous or aborigin* or “torres strait” or MH “Oceanic Ancestry Group”). Limited to English language. See Additional file 1 for full details of the search strategy.

### Study selection, data extraction and quality assessment

**Fig. 1:**
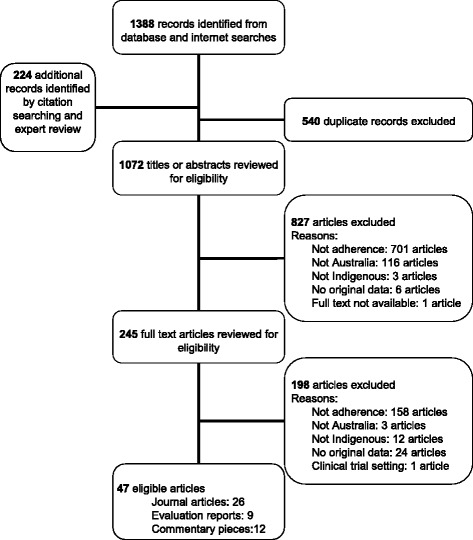
Flowchart of literature review results and study selection procedure

Data elements extracted were: study aim, methodology, site, dates; participant eligibility criteria; recruitment strategy; sample size; data collection method; data analysis strategy; results. Study quality was assessed using a tool adapted from McInnes and Chambers [10] (see Additional file 2). The study quality criteria were not applied to evaluation reports, correspondence or commentary pieces. Due to the limited data available on the subject, study quality score was not used to exclude articles or weight study findings. Data extraction and quality assessment were completed by the first author. Findings from qualitative studies were compiled in a narrative synthesis. See Fig. 1 below for a flowchart of the article selection process.

## Results

The 47 included articles reported on people living with: HIV, diabetes, epilepsy, kidney disease, mental health issues, tuberculosis, cancer, cardiovascular disease and those who had experienced a seizure. Five sources reported rates of adherence and 14 reported barriers, facilitators or strategies, either from the patient or health professional perspective. Participants from all states and territories except the Australian Capital Territory and Tasmania were represented. The characteristics, results and quality scores for the journal articles are included in Table 1 below (when multiple articles reported on the same study the information has been combined). Summaries of the evaluation reports and letter in reply can be found at Additional file 3; commentary pieces were not summarised.

**Table 1:** Characteristics and findings of included journal articles

Reference	Study aim	Study population/Participants	Setting	Study design & data collection method	Sample size	Key findings	Quality score
[32]	To explore patients’ understanding of end stage kidney disease	Patients with end stage renal disease, health professionals (HPs) and other relevant people	9 hospital renal units & 17 dialysis centres from NSW, WA, QLD, SA & NT	Interviews	241 patients (incl 146 Indigenous people)	One patient indicated he didn’t take medicines because he was busy doing ‘cultural stuff’	50%
[22]	To identify social and cultural issues affecting kidney transplants, compliance and transplant outcomes	Aboriginal and/or Torres Strait Islander people who received kidney transplant between October 1983 and February 1994 and HPs from a major referral hospital	Patients who attended Princess Alexandra Hospital, Brisbane, QLD	Medical record review & in depth interviews	11 patients (number of HPs not provided)	One patient linked their graft rejection to poor adherence to medicine. HPs indicated inadequate adherence was an issue; they were frustrated when their efforts to address adherence were not followed by changes in patient behaviour.	18%
[12]	To identify the characteristics and outcomes of Aboriginal people with Type 2 diabetes mellitus (T2DM)	Urban dwelling Aboriginal people with T2DM	Fremantle, WA	Self-reported adherence measured using standardised questionnaires	1312 (incl 18 Aboriginal patients)	42% of Aboriginal patients reported missing doses occasionally or regularly (compared with 20% of Anglo-Celt patients) (*p* = 0.07).	92%
[40]	To explore medication use by older women	Women approximately 60 years old	Adelaide, SA	Semi structured interviews	140 (incl 12 Aboriginal women)	Barriers to adherence: sharing medicines; stopping medicines when they felt better; forgetting to take doses. Suggested strategy: development of culturally appropriate medicine education resources.	36%
[41]	To evaluate the uptake and outcomes of a cardiac rehabilitation program	Patients who attended Heart Health program	Metropolitan Aboriginal Medical Service, WA	Interviews, questionnaires, yarning sessions & assessment of risk factors	Not reported	Adherence: some patients indicated they took medicine inconsistently. Barriers to adherence: sharing medicines; taking expired medicines.	39%
[44]	To explore the interface of Warlpiri culture and identity with biomedical elements of T2DM	People living with T2DM and their family members.	Remote Central Australian community, NT	Interviews	84 people with T2DM, 14 family members	Barriers to adherence: forgetting medicines while travelling; clinic not providing sufficient medicines to cover duration of trip; difficulty accessing medicines away from primary clinic; belief in God which meant one participant did not believe she need to take medicine.	21%
[30]	To explore issues faced by Indigenous people with mental health issues, carers and family members	Indigenous people with mental health issues, carers and family members	Urban, regional and remote areas, SA	Interviews & focus groups	130	Barriers to adherence: low English literacy; competing priorities; cost of medicines; no safe storage for medicines at home; swapping medicines. Suggested strategy: racism needs to be eliminated from health services. HP perspective: one indicated that some patients ‘just didn’t care’ about being adherent.	75%
[53]	To explore why people presented late for treatment of tuberculosis (TB), and explore issues with adherence	Aboriginal community members, HPs, council employee	Remote Top End community, NT	Interviews, focus groups, (conducted in English) & observations	51 (18 individual interviews, 5 focus groups)	Barriers to adherence: low level of perceived risk of latent TB; HP reported that some patients believed in the power of the mind, and therefore did not take medicine; limited clinic opening hours prevented accessing medicines. Authors suggest the long duration of treatment and side effects may impair adherence. Suggested strategy: increase the involvement of Aboriginal Health Practitioners (AHPs).	14%
[29]	Explore the use of medicines by Indigenous people from the perspective of Aboriginal Health Workers (AHW)	Aboriginal Health Workers	Community health centres & hospitals, mid western NSW	In depth interviews	11	Attitude to adherence: some said taking medicine was ‘not cultural’. Barriers to adherence: communication barrier between HP and patient; low literacy; sharing medicines. Suggested strategies: involvement of AHWs in medicine management; cultural awareness training for pharmacists.	68%
[14]	To evaluate a chronic disease program	Community residents with risk factors for chronic disease	Remote Top End community, NT	2001–2003 medicine adherence captured using clinical audit	264	In 1996–98 2/3 of participants reported taking medicines ‘some or most of the time’ (data collection method not reported). In 2001–03 ~70% of prescribed medicines were being collected from the pharmacy. Authors attribute deterioration in clinical outcomes to reduction in compliance (evidence of reduced compliance was provided as a personal communication by the pharmacist).	Could not be assessed
[28]	To explore HPs’ experiences and attitudes towards adherence in Indigenous health	HPs working in the NT	4 hospitals, 2 Aboriginal Medical Services and some Department of Health programmes, NT	Pre interview question sheet, focus groups	76	97% HPs reported that ‘non compliance’ was a major or significant problem. 3 most frequently reported barriers to adherence: inadequate communication between HP and patient; inadequate cross cultural training of HPs; insufficient numbers of AHPs. 3 most commonly reported facilitators of adherence: an understanding of Western medicine; family support; good rapport between HP and patient.	39%
[15]	To investigate characteristics of Indigenous Australians with poorly controlled T2DM	Indigenous people, 18–65 years, with HbA1c ≥ 8.5%	12 clinics, rural north QLD	Method for measuring adherence not reported	193	46% of Aboriginal participants were adherent to all medicines; 31% of Torres Strait Islander participants were adherent to all medicines. Authors suggest inadequate dose titration and clinical inertia contributed to poor clinical outcomes.	96%
[18]	To evaluate outcomes of Aboriginal patients after open heart surgery	Aboriginal people who had open heart surgery between July 1996 and November 2001	Sir Charles Gairdner Hospital, WA	Clinical record review & telephone follow up (method of measuring adherence not reported)	57	Authors report that three patients were ‘irregular’ with their anticoagulation medicines (data source unclear). Authors assert that compliance in patients who could not be contacted was ‘likely to be low’. Poor clinical outcomes and one death were attributed to poor compliance by the authors.	46%
[34]	To identify barriers to providing culturally appropriate services to Aboriginal people with diabetes	HPs working with Aboriginal people with T2DM	Government administered health regions, SA	Semi structured questionnaire	43	Barrier to adherence: patients did not consider T2DM a priority (they had other more pressing issues to manage)	36%
[27] & [37]	To explore experiences of health professionals working with Aboriginal people with mental health issues	HPs working with Aboriginal and Torres Strait Islander people with mental health issues	Urban, regional and remote areas, SA	Survey	114	39% health professionals reported compliance was an issue. Most commonly reported barriers to adherence: sharing medicines; side effects; cost of medicines; not travelling with medicines. Facilitators of adherence: DAAs; supporting patients to resolve broader life issues.	75%
[21]	To explore barriers to mental health service delivery in remote communities	HPs working in mental health in remote areas	Remote primary health centres, NT	Semi structured interviews	41	82.9% HPs said non adherence was a common cause of relapse. 87.2% HPs reported that poor compliance was a barrier to prescribing oral medicines.	−4%
[70]	To explore HPs’ perspectives of the experience of Aboriginal people with cancer	HPs providing cancer services to Aboriginal people	Metropolitan and remote locations, WA	In depth interviews	62	Suggested strategy: Aboriginal liaison officers or cancer nurse coordinators should repeat medical information to patients after they have seen the clinician	61%
[19]	To document epidemiology of tuberculosis cases	All cases of tuberculosis notified from January 1993 – December 1997	Far North Queensland	Medical record review	87 (including 50 Aboriginal and/or Torres Strait Islander people)	All relapses occurred in Aboriginal and/or Torres Strait Islander patients; all had documented ‘compliance problems, mainly attributed to alcohol abuse’. Facilitator of adherence (as reported by authors): increased employment of AHPs.	36%
[23]	To determine the readiness of community pharmacists to play a larger role in Indigenous health	Community pharmacists working in areas with significant Indigenous populations	Urban, rural and remote NSW	Semi structured in depth interviews	27	Attitudes towards adherence: participants felt that adherence was a major problem, and one stated that ‘they’re very poor tablet takers’. Suggested strategies: DAAs; involvement of AHPs in dispensing; cultural training for HPs; development of culturally appropriate resources.	64%
[33]	Explore experiences of Aboriginal and/or Torres Strait Islander people with medicines	Aboriginal people taking multiple medicines	Primary health centres in urban, rural and regional QLD, NT, SA, NSW and VIC	Semi structured focus groups (conducted in English)	101	Barriers to adherence: difficulty accessing medicines while travelling; forgetting; fear of Western medicine; other more pressing issues; change in tablet appearance; information provided difficult to read and understand. Facilitators of adherence: DAAs; reminders from families and friends. Suggested strategy: provision of more information.	54%
[45] & [46]	To explore perspective of Torres Strait Islander people with diabetes	Torres Strait Islander people with T2DM	8 remote communities, Torres Strait Islands	In depth interviews, focus groups	67	Barriers to adherence: forgetting; side effects; lack of family support; not wanting to feel like a diabetic (one person); some participants refused to take medicines, saying that they believed in God. Suggested strategy: support groups. Authors suggest that limited understanding of how medicines worked was likely to impair adherence and therefore clearer information was required.	57%
[49]	To explore the role of alcohol in the lives of Aboriginal people with HIV	Aboriginal people who were HIV +	Metropolitan and rural areas, WA	Semi structured interviews	20	Barrier to adherence: alcohol intake. Facilitators of adherence: Reducing intake of alcohol; waiting until all medicines taken for the day before starting drinking alcohol.	89%
[39]	To explore perceptions of financial burden associated with chronic condition medicines	People with a chronic condition or their carer	Regional QLD, WA & NSW	Semi structured in depth interviews	97 (incl 23 Aboriginal and/or Torres Strait Islander)	Barrier to adherence: some indicated cost was an issue, but most reported there was no cost for their medicines (they were covered by the Closing the Gap subsidy program).	79%
[13]	To assess the contributions of alcohol, head trauma and medicine adherence to hospital presentations for seizure	People presenting to hospital with a seizure between 19 October 2006 and 30 December 2007	Cairns Base Hospital, QLD	Medical record review & questionnaire	127 (incl 26 Indigenous patients)	Self reported adherence for Indigenous Australians: −35% never missed medicines; −18% missed <2 times/month; −0% missed >2 times/month; −29% missed at least 2 times/week; −18% hardly ever take/never take medicines. Indigenous Australians were less likely to take medicines than non-Indigenous Australians (*p* < 0.05). Suggested strategies (by authors) to enhance adherence: once daily dosing; prescription of medicines with the least side effects.	75%

*Abbreviations*: *AHP* Aboriginal health practitioner, *HP* Health professional, *T2DM* Type 2 diabetes mellitus, *TB* Tuberculosis

Study quality scores ranged from −4 to +96% (median: 54; IQR: 39). The criteria could not be applied to one study due to the absence of any methodological information on the assessment of adherence [11]. The most common issue was incomplete methodology reporting.

### Adherence rates

Six articles included quantified adherence rates and these studies reported that approximately two thirds of Indigenous Australians take their regular medications at least some of the time (rates and definitions from individual studies are listed in Table 1) [12–17]. The methods used to quantify adherence were rarely reported in detail and studies differed in the way they quantified or categorised adherence; consequently a meta-analysis was not possible and comparison of findings was difficult.

Two of these studies found that Indigenous Australians were less adherent than non-Indigenous Australians [12, 13]. These findings were based on self-reported adherence. and we did not identify any studies validating self-reported adherence in this population so the accuracy of the data is unclear.

### Outcomes of adherence

Few studies have explored associations between adherence and clinical outcome, or sought Indigenous Australians’ views on this relationship. Some authors attributed poor clinical outcomes (including relapse of tuberculosis; failure of a kidney graft; recurrence of angina after heart surgery; and death after heart surgery) to inadequate adherence [18–20] and 82.9% of health professionals working in mental health services in the NT reported inadequate adherence as a common cause of relapse [21]. Predictors of clinical outcome are multifactorial, for example, poor clinical outcomes in Indigenous Australians with diabetes have been attributed to clinical inertia and inadequate dose titration rather than adherence problems [15].

In a study of cultural beliefs of disease causation (specifically, cultural factors affecting renal transplant outcome), just one patient connected their inadequate adherence to a negative health outcome (graft rejection); other patients instead nominated factors such as alcohol, poor nutrition and separation from kin and country as causes of poor health outcomes [22].

### Attitudes about adherence rates

In the majority of studies, health professionals expressed the view that Indigenous Australians have inadequate adherence to medications [23, 24] and this is having a negative impact on Indigenous health in Australia [22, 24–27]. In one sample 97% of health professionals believed it was a ‘major or significant’ problem [28]. Just one study reported a positive view of adherence, with an Aboriginal Health Practitioner saying that ‘a good percentage are compliant’ [25].

Many health professionals acknowledged the challenges faced by Indigenous Australians taking long-term medicines, but there were mixed views on patients’ attitudes to medicines and adherence [29–31]. No studies reported patients’ own perceptions of their adherence.

### Barriers to adherence

The literature reports numerous challenges experienced by Indigenous Australians requiring long-term medicines, with much agreement between providers and patients. Barriers reported by both patients and health professionals were: having other priorities including sociocultural obligations which were more important than taking medicines and often involved travelling away from their community; [32–36] cost; [25, 27, 30, 37–39] sharing or swapping medicines; [27, 29, 30, 37, 40–42] stopping medicines once feeling better [25, 40] and issues obtaining medicines while away from home [33, 43, 44].

An issue reported by patients only was forgetting to take doses [31, 40, 45, 46] and a few participants stated that their belief in God meant they did not need to take medicines [44–46]. In addition, health professionals reported that inadequate safe storage for medicines at home impaired adherence [30, 47].

### Enablers of adherence

Dose administration aids (DAAs) and other simple strategies have been associated with improved adherence for Indigenous Australians. Patients in two studies reported that DAAs were useful, [33, 48] and in a small (*n* = 11) Central Australian study, the provision of DAAs increased patient adherence from 70% to 87% (*p* = 0.019). [16] Patients with HIV indicated that adjusting quantity/timing of alcohol consumption assisted with adherence, [49] and patients with diabetes indicated once-daily dosing assisted [48]. Health professionals reported that a good understanding of Western medicine and establishing good rapport with patients was associated with good adherence [28].

Approaches which engaged communities, and involved Aboriginal Health Practitioners and family, appeared to improve adherence. For example, increased availability of Aboriginal Health Practitioners was associated with higher adherence to tuberculosis treatment in northern Queensland [19] and was part of a successful strategy implemented at an Indigenous Health Service in Brisbane [36]. The involvement of community members in medication dispensing in a remote NT community was also associated with increased adherence [43]. Both health professionals and patients reported that family support enhanced adherence [28, 33].

Medication cost reductions via the Australian Pharmaceutical Benefits Scheme co-payment, have alleviated the financial burden and reportedly improved adherence [43, 50].

### Suggested strategies to improve adherence

The health professionals and participants interviewed in the eligible studies suggested a variety of approaches which they felt would enhance adherence; the proposed strategies aligned well with the barriers and facilitators described. Both groups felt that culturally appropriate resources designed to enhance the provision of patient education about medicines would increase adherence [17, 24, 33, 40, 51, 52]. Patients also suggested the establishment of a support group [45, 46]. Health professionals proffered a variety of adherence support strategies: increased involvement of Aboriginal Health Practitioners in medication management; [24, 52–54] simplification of dose regimens, including once-daily dosing and long acting formulations; [17, 35, 36, 52, 54–56] provision of DAAs, [27, 37, 52, 55] and the use of home medicines reviews [24, 42, 51] (although adaptation of this program is required to suit the needs of Indigenous Australians [25]). Some health professionals also emphasised the need to address social determinants of health [17, 27, 37, 54].

A summary of the opportunities to improve adherence is provided in Table 2.

**Table 2:** Opportunities to improve adherence

		References
Proven strategy	• Dose administration aid (increased adherence from 70% to 87%)	[16]
Facilitators reported by patients and health professionals	• Family support	[28, 33]
Facilitators reported by patients	• Dose administration aids • Adjusting alcohol use • Once daily dosing	[33, 48] [49] [48]
Facilitators reported by health professionals	• Establishing good rapport with patients • Patients having a good understanding of Western medicine • Involvement of Aboriginal Health Practitioners (AHPs) • Involvement of community members in dispensing	[28] [28] [19] [36] [43]
Strategies suggested by patients and health professionals	• Development of culturally appropriate education resources	[17, 24, 33, 40, 51, 52]
Strategy suggested by patients	• Support group	[45, 46]
Strategies suggested by health professionals	• Increased involvement of AHPs in medicine management • Simplification of dose regimens • Home medicines reviews • Address social determinants of health	[24, 52–54] [17, 35, 36, 52, 54–56] [24, 42, 51] [17, 27, 37, 54]

## Discussion

We identified a range of publications addressing adherence among Indigenous Australians. The articles varied widely in research methodology and quality, preventing a meta-analysis, and some of the sample sizes were small (two studies each with 11 participants). However, despite differences in study design and quality, many findings were remarkably similar, indicating the value of including all eligible studies for this review.

### Adherence rates

Health professionals were essentially unanimous in seeing inadequate adherence as an important issue in Indigenous health in Australia, but just two studies reported results which support the assertion that adherence for Indigenous Australians is significantly worse than for non-Indigenous Australians [12, 13]. A number of studies found that two thirds of Indigenous Australians were adherent (based on varying definitions of ‘adherence’) [14, 16, 17], which aligns with findings of international studies across a range of populations and health conditions [57]. The potential discrepancy between provider beliefs and reality requires clarification through further studies using validated methods to measure adherence.

### Barriers and facilitators of adherence

There was considerable overlap in the barriers and facilitators reported by health professionals and Indigenous Australians living with chronic conditions, indicating that many health professionals are aware of the challenges faced by their patients. Some patients reported that traditional or religious beliefs could cause or prevent ill health [22, 44–46] supporting the call for improved health literacy made by consumers and practitioners working in Indigenous health in Australia [17, 24, 33, 40, 51, 52]. Some very context-specific barriers were identified, such as adherence challenges for remote-dwelling Indigenous Australians during travel away from home communities [32–36], however many barriers to adherence faced by Indigenous Australians are universal. Forgetting doses, complex dosing schedules, the cost of medicines, inadequate social support and alcohol use were all identified as key factors in a 2008 international review of adherence [58]. This study also found that minority groups had poorer compliance, but concluded that socio-economic status was more likely to explain lower compliance than race [58].

### Strategies to enhance adherence

The studies provided a range of suggested and proven adherence support strategies relevant for Indigenous Australians however there was only one example of a strategy being tested and evaluated in a research study (see Additional file 3 for details) [16]. Two other studies suggest there could be further scope for pill burden minimisation for Indigenous Australians. The Kanyini GAP study (not included in this review because results were not disaggregated by ethnicity) showed that dose regimen simplification (through the use of a polypill) can enhance adherence in a population with a large proportion of Indigenous Australians [59, 60]. Dose simplification also improved adherence in a New Zealand study which included a large proportion (50%) of Maori participants [61].

Other intervention studies testing adherence support strategies for Indigenous Australians have been published [62–65] or are being undertaken [66]; they provide relevant insights such as the value of directly-observed treatment, but did not meet the selection criteria for this review.

### Specificity of findings

Some of the findings reported in this review may be specific to the study context – findings from remote settings for instance should not be extrapolated to Indigenous Australians living in urban areas, however it is worthwhile highlighting the consistency in information reported which was perhaps unexpected given the diversity of participant groups, which varied by condition, location, sex and age. Our findings are supported by previous non-systematic reviews of adherence among Indigenous Australians [5, 6].

### Remaining evidence gaps

Unfortunately few papers accurately quantified adherence and just five articles linked adherence to clinical outcomes (none of which reported any statistical results). Consequently the size of the problem, the validity of health professional perspectives and the potential gains which could be achieved by focusing on adherence support strategies remain unknown. In addition, very limited information has been collected from Indigenous Australians who live in remote communities and those who do not speak English fluently; so further research is required to capture their views [67].

While many solutions can be identified from the international literature, specific locally-relevant strategies are also needed. It is heartening to see some translation of research findings into practice; the 2005 National Health and Medical Research Council guidelines for strengthening cardiac rehabilitation of Indigenous Australians includes evidence-based strategies for adherence support [68]. Continuing concerns about adherence and the ongoing significant burden caused by cardiovascular disease [69] and the absence of evidence-based recommendations for the management of other chronic conditions indicate more needs to be done.

We need more evidence on which activities effectively support Indigenous Australians requiring long-term medicines. This review indicates we have the necessary information to develop tailored, locally-relevant strategies.

### Strengths and limitations

The key strengths of this review are the inclusion of qualitative and quantitative studies, the inclusion of grey literature and the use of a quality assessment tool. A limitation of this study is that initial study selection, data extraction and quality assessment were conducted by one individual (J.L.dD), however ongoing input from researchers with experience in systematic literature reviews and chronic conditions ensured the rigour of the implementation of the methodology and the results.

## Conclusions

‘Closing the gap’ in health outcomes for Indigenous Australians with chronic conditions requires lifestyle modification, changes in health-seeking behaviour and adherence support. Data on adherence rates are limited, but it is likely that, as is the case in many patient populations, suboptimal adherence means that Indigenous Australians are not receiving the full benefit of medications. The maximum benefit of medications for chronic conditions is obtained with 100% adherence and in some chronic conditions such as HIV and rheumatic heart disease, missed or late doses can be particularly harmful. Therefore the target adherence for all people living with chronic conditions should be 100%.

This review informs clinical practice in several key ways. Clinicians who presume low adherence among Indigenous Australian patients, and may choose not to prescribe certain medications based on this presumption, need to acknowledge that this belief is not evidence-based. Clinicians prescribing for Indigenous Australians need to utilise methods which improve medication adherence, including dose administration aids. Additionally, methods which Indigenous Australians have requested or practitioners have suggested (Table 2) should be incorporated, including involving Indigenous health practitioners, using family- and community-centred approaches, and culturally-appropriate educational resources to achieve good rapport.

This review also informs future research priorities. Rigorous measurement and reporting of adherence using standard measures is needed and this could be achieved by using routinely collected dispensing data to calculate the medication possession ratio (an indicator widely used in adherence research). Randomised controlled trials of interventions, incorporating strategies such as those described herein would provide the robust data needed in this field to be able to improve adherence and therefore improve outcomes among people with chronic conditions.

In addition to gathering adherence data, existing information on facilitators should be used to develop, implement and evaluate interventions. Policy makers and health service managers should allocate appropriate resources for program delivery and evaluation and the findings should be published with detailed methodology information to maximise the evidence base.

Finally, the term ‘adherence’ itself may not be ideal, since it fails to acknowledge the partnership required to optimise chronic condition management. The focus should be on the development and maintenance of a respectful, trusting relationship between patient and health professional, one that is not overshadowed by assumptions of poor adherence.

## Additional files

Additional file 1: Search strategy details. Full details of the search strategy are provided. (DOC 52 kb)

Additional file 2: Quality assessment tool. The quality assessment template including scoring instructions. (DOC 60 kb)

Additional file 3: Table: Characteristics and findings of evaluation reports and correspondence. A summary of the relevant information extracted from evaluation reports and correspondence. (DOC 73 kb)
