# Supplementary material for: A systematic review of adherence in Indigenous Australians: an opportunity to improve chronic condition management
Source: BMC Health Serv Res. 2017 Dec 27;17:845. doi: 10.1186/s12913-017-2794-y (PMC5745645; doi:10.1186/s12913-017-2794-y)
Supplement: Supplementary file 1 — Search strategy details. Full details of the search strategy are provided. (DOC 52 kb) [file 12913_2017_2794_MOESM1_ESM.doc]

**Additional file 1:** Search strategy

| **Databases** | **Search terms** |
| --- | --- |
| Academic Search Premier;CINAHL Plus with Full Text;Health Source: Nursing/Academic Edition;MEDLINE with Full Text;PsycINFO | (((*adheren* OR *complian* OR concord*) and (treatment* or medicine* OR medication* OR drug*)) or (MM "Patient Compliance+" OR MM "Medication Adherence" or DE "PATIENT compliance" or DE "Treatment Compliance")) and (indigenous or aborigin* or "torres strait" or MH "Oceanic Ancestry Group" or DE "Indigenous Populations" or DE "ABORIGINAL Australians" OR DE "WOMEN, Aboriginal Australian" OR DE "CHILDREN, Aboriginal Australian" OR DE "BOYS, Aboriginal Australian" or DE "FAMILIES, Aboriginal Australian" or DE "INDIGENOUS peoples") |
| Greylit.org | (indigenous or aboriginal) and (complian* or adheren*) |
| Australian Indigenous Healthinfonet | (adheren* or complian*) |

‘*’ Indicates truncation
