# Supplementary material for: A systematic review of adherence in Indigenous Australians: an opportunity to improve chronic condition management
Source: BMC Health Serv Res. 2017 Dec 27;17:845. doi: 10.1186/s12913-017-2794-y (PMC5745645; doi:10.1186/s12913-017-2794-y)
Supplement: Supplementary file 2 — Quality assessment tool. The quality assessment template including scoring instructions. (DOC 60 kb) [file 12913_2017_2794_MOESM2_ESM.doc]

**Additional file 2: Quality assessment tool**

| **Methodology quality assessment criteria** | **Fully met** | **Partially met** | **Not met** | **Not applicable** |
| --- | --- | --- | --- | --- |
| Clear aims/research question |  |  |  |  |
| Design appropriate to answer research question |  |  |  |  |
| Setting appropriate for research question |  |  |  |  |
| Recruitment process given |  |  |  |  |
| Participants appropriate to research question |  |  |  |  |
| Ethical approval/informed consent given |  |  |  |  |
| Data collection method adequately described |  |  |  |  |
| Relationship between the researchers and the participants described? |  |  |  |  |
| Clear description of analytical method |  |  |  |  |
| Analysis not biased by researcher (more than one analyst, triangulation, checked with participants) |  |  |  |  |
| Findings presented in sufficient detail |  |  |  |  |
| Findings discussed in context |  |  |  |  |
| Implications discussed |  |  |  |  |
| Limitations of study discussed |  |  |  |  |

In order to assess the methodological quality of each study, items were scored as follows:

- Items identified as ‘fully met’ were given a score of +1
- Items identified as ‘partially met’ were given a score of +0·5
- Items identified as ‘not met’ were given a score of –1
- Items identified as ‘not applicable’ were scored as zero.

The scores of all applicable items were then totalled, and the percentage score was derived by dividing the total by the number of applicable items.

(adapted from McInnes RJ, Chambers JA. Supporting breastfeeding mothers: qualitative synthesis. J Adv Nurs. 2008 May;62(4):407-27. doi: 10.1111/j.1365-2648.2008.04618.x)
