# Supplementary material for: A systematic review of adherence in Indigenous Australians: an opportunity to improve chronic condition management
Source: BMC Health Serv Res. 2017 Dec 27;17:845. doi: 10.1186/s12913-017-2794-y (PMC5745645; doi:10.1186/s12913-017-2794-y)
Supplement: Supplementary file 3 — Table: Characteristics and findings of evaluation reports and correspondence. A summary of the relevant information extracted from evaluation reports and correspondence. (DOC 73 kb) [file 12913_2017_2794_MOESM3_ESM.doc]

**Supplementary File 3:**

**Table: Characteristics and findings** of evaluation reports and correspondence

| **Reference** | **Document type** | **Study aim/report purpose** | **Study population/**  **Participants** | **Setting** | **Data collection method** | **Sample size** | **Key findings** |
| --- | --- | --- | --- | --- | --- | --- | --- |
| **[1]** | Report | Evaluation of the Indigenous Chronic Disease Package | Interviews: health professionals from Aboriginal Health Services; stakeholders from state and national level health organisations; pharmacists. Focus groups: community members from case study sites. | Nationwide (urban, region and remote) | Focus groups, interviews, clinic data, Government indicator datasets | Interviews: more than 700; focus groups: 670 participants; clinic data: 41 health services. | 88% of clinicians agreed the PBS co-payment has improved adherence.  Increased access to medicines means people have more medicines to manage, and appropriate support (eg: HMRs) is now even more important. |
| **[2]** | Report | Final report of the Home Medicines Review qualitative research project | Consultations: Key stakeholders; interviews: health professionals, consumers and carers; focus groups: consumers | Stakeholder consultations: nationwide; interviews: 8 divisions of general practice (NSW, VIC, QLD, WA, SA & TAS); focus groups: 10 divisions of general practice (NSW, VIC, QLD, WA, SA & TAS); | Public submissions, stakeholder consultations, interviews, focus groups | Public submissions: 84; interviews: 178; focus groups: 100 participants | Health professionals concerned by non adherence by Indigenous people and the resulting hospitalisations.  Barriers to adherence: cost of medicines; thinking that medicine isn’t needed once person is feeling better; Suggested strategies: HMR program requires numerous adaptations to suit Aboriginal people. |
| **[3]** | Report | Comparison of a tablet safe with dose administration aids (DAAs) as facilitators of adherence | Aboriginal and/or Torres Strait Islander people over 15 years old, living in the study community, prescribed at least two medicines for chronic condition management. | Small remote Central Australian community | Adherence measured by pill counts and researcher administered questionnaires | 25 (11 in DAA group, 14 in tablet safe group) | Baseline adherence for DAA group: 70%; for tablet safe group: 73%.  After 4 weeks, DAA group had increased adherence to 87% (p=0.019); no significant change to adherence in tablet safe group (66%, p not reported).  All tablet safe group wanted to keep the safe after the trial. |
| **[4]** | Report | Evaluation of PBS medicine supply arrangements for remote Aboriginal Health Services | Public submissions: health organisations, consultant pharmacist.  Case studies: Health professionals working at remote health services, Survey: pharmacists (remote and hospital based)  Interviews: key stakeholders from national and state level health organisations | Stakeholder interviews, quantitative data and survey: nationwide; case studies: WA, NT, SA, QLD, NSW. | Public submissions, interviews, surveys, case studies, existing quantitative data on medicine utilisation and expenditure | Public submissions: 9;  survey: 88; case studies: 13 | Barriers to adherence: filling scripts at a different health service.  Facilitators of adherence: subsidisation of medicine costs; involvement of local community members in dispensing. |
| **[5]** | Report | Evaluation of the Closing the Gap medicine subsidy scheme | Health professionals, stakeholders, Aboriginal and/or Torres Strait Islander community members, | Stakeholders consulted from organisations at national and state level (incl NT); community visits to 8 sites (one in each state and the NT). | Regional forums, focus groups and interviews, case studies, analysis of existing quantitative data | ~88 staff and 131 community members consulted during site visits.  N for stakeholder consultations and regional forums not reported. | Barrier to adherence: sharing medicines (which may have increased with the introduction of subsidy programs which only assist some patients)  Suggested strategies (from pharmacists): home medicine reviews, dose administration aids and patient education |
| **[6]** | General correspondence published in journal | Identification of the barriers to adherence | Indigenous people with T2DM | Urban South Brisbane, QLD | Medical record review, survey (self reported adherence) | 82 | 39% reported poor adherence. Facilitators: once daily dosing; dose administration aids |
| **[7]** |  | To re-define the Aboriginal health problem and learn from Aboriginal people how health service delivery by the Central Australian Aboriginal Congress could be improved. | Community members and health professionals | 8 communities in Central Australia | Group discussions and interviews | Not reported | Some people sought assistance from the *ngangkere* (a traditional healer), but sought Western medicine for ‘white man’s disease’ which included many chronic conditions.  Facilitator of adherence: delivery of medicines to people’s homes.  Barrier to adherence: not enough medicines provided at each occasion (people wanted at least one week’s supply) |
| **[8]** | Unpublished report | Annual report of the Australia and New Zealand dialysis and transplant registry (ANZDATA) | Aboriginal patients receiving dialysis or kidney transplant between 1983 and 1988 | SA and NT | Data from ANZDATA database | 37 | Authors attribute graft failure in part to non adherence to anti-rejection medicines |
| **[9]** | Report | Evaluation of the home medicine review (HMR) program at Pika Wiya Aboriginal Health Service | Medical record review: Patients who received HMR.  Questionnaires: General practitioners (GPs), Aboriginal Health Practitioners (AHPs) and pharmacy staff | Aboriginal Health Service, WA | Medical record review, questionnaires | Record review: 95 HMR reviews  Questionnaire: 4 GPs, 8 AHPs (n for pharmacy staff not reported) | Barriers to adherence: other competing issues in patient’s life (eg: housing); side effects. Adherence issues were the second most common reason for a referral to the HMR program. Suggested strategies: simplified dosing regimens; dose timings tailored to individual’s circumstances; avoid brand changes. |

**References:**

1. Bailie R, Griffin J, Kelaher M, McNeair T, Percival N, Laycock A, et al. Sentinel Sites Evaluation: Final Report. Canberra: Australian Government Department of Health and Ageing, 2013.

2. Campbell Research & Consulting. Home Medicines Review Program Qualitative Research Project - Final Report Victoria Campbell Research & Consulting,, 2008.

3. Davis R, Scholz A, Muller R, Charlesworth K. Evaluation of Two Interventions to Assist Medication Concordance in a Remote Aboriginal Community. A Report for the National Prescribing Service. National Prescribing Service, 2002.

4. Kelaher M, Taylor-Thompson D, Harrison N, O'Donoghue L, Dunt D, Barnes T, et al. Evaluation of PBS Medicine Supply Arrangements for Remote Area Aboriginal Health Services Under SECTION 100 of the National Health Act. Cooperative Research Centre for Aboriginal and Tropical Health, Menzies School of Health Research and the Program Evaluation Unit, University of Melbourne, 2004.

5. KPMG. National monitoring and evaluation of the Indigenous chronic disease package:First monitoring report 2010-11. Canberra: Australian Government Department of Health, 2013.

6. Morton AP. Characteristics and outcome of type 2 diabetes in urban Aboriginal people [3]. Intern Med J. 2007;37(8):581-2.

7. Nathan P. Health business: Heinemann Educational Australia; 1983.

8. Pugsley D. Dialysis and transplantation in the Aboriginal population of South Australia and the Northern Territory. Adelaide: Queen Elizabeth Hospital, 1989.

9. Sanburg A. An Evaluation of the Home Medicines Review (HMR) process at the Pika Wiya Aboriginal Health Service (PWHS). South Australia: RGH Pharmacy Consulting Services Pty Ltd, 2009.
